# The GLA:D® Canada program for knee and hip osteoarthritis: A comprehensive profile of program participants from 2017 to 2022

**DOI:** 10.1371/journal.pone.0289645

**Published:** 2023-08-03

**Authors:** James J. Young, Anthony V. Perruccio, Christian J. H. Veillette, Rhona A. McGlasson, Michael G. Zywiel

**Affiliations:** 1 Schroeder Arthritis Institute, Krembil Research Institute, University Health Network, Toronto, Ontario, Canada; 2 Center for Muscle and Joint Health, Department of Sports Science and Clinical Biomechanics, University of Southern Denmark, Odense, Denmark; 3 Institute of Health Policy, Management and Evaluation, Dalla Lana School of Public Health, University of Toronto, Toronto, Ontario, Canada; 4 Department of Surgery, Faculty of Medicine, University of Toronto, Toronto, Ontario, Canada; 5 Bone and Joint Canada, Canadian Orthopaedic Foundation, Toronto, Ontario, Canada; Mugla Sitki Kocman Universitesi, TURKEY

## Abstract

**Background:**

The Good Life with osteoArthritis in Denmark (GLA:D®) program was implemented in Canada in 2017 with the aim of making treatment guideline-recommended care available to the 4 million Canadians with knee and hip osteoarthritis (OA). This report describes the GLA:D® Canada program, registry and data collection procedures, and summarizes the sociodemographic and clinical profile of participants with knee and hip OA to inform the scientific research community of the availability of these data for future investigations and collaborations.

**Methods:**

The GLA:D® program consists of three standardized components: a training course for health care providers, a group-based patient education and exercise therapy program, and a participant data registry. Patients seeking care for knee or hip OA symptoms and enrolling in GLA:D® are given the option to provide data to the GLA:D® Canada registry. Participants agreeing to provide data complete a pre-program survey and are followed up after 3-, and 12-months. Data collected on the pre-program and follow-up surveys include sociodemographic factors, clinical characteristics, health status measures, and objective physical function tests. These variables were selected to capture information across relevant health constructs and for future research investigations.

**Results:**

At 2022 year-end, a total of 15,193 (11,228 knee; 3,965 hip) participants were included in the GLA:D® Canada registry with 7,527 (knee; 67.0%) and 2,798 (hip; 70.6%) providing pre-program data. Participants were 66 years of age on average, predominately female, and overweight or obese. Typically, participants had knee or hip problems for multiple years prior to initiating GLA:D®, multiple symptomatic knee and hip joints, and at least one medical comorbidity. Before starting the program, the average pain intensity was 5 out of 10, with approximately 2 out of 3 participants using pain medication and 1 in 3 participants reporting a desire to have joint surgery. Likewise, 9 out 10 participants report having previously been given a diagnosis of OA, with 9 out 10 also reporting having had a radiograph, of which approximately 87% reported the radiograph showed signs of OA.

**Conclusion:**

We have described the GLA:D® Canada program, registry and data collection procedures, and provided a detailed summary to date of the profiles of participants with knee and hip OA. These individual participant data have the potential to be linked with local health administrative data registries and comparatively assessed with other international GLA:D® registries. Researchers are invited to make use of these rich datasets and participate in collaborative endeavours to tackle questions of Canadian and global importance for a large and growing clinical population of individuals with hip and knee OA.

## Introduction

Over 300 million individuals worldwide have symptomatic knee and hip osteoarthritis (OA) [1] and the Public Health Agency of Canada estimates approximately 14% of the adult population (4 million Canadians) live with diagnosed OA [2]. Across global health systems the cost of OA is estimated to be 1% of total healthcare expenditures and 0.5% of Gross Domestic Product [3]. For example, OA accounts for 2.6% (80 billion USD) of total health care spending in the United States [4] and over 1.3 billion CAD is spent on knee and hip arthroplasties alone in Canada [5]. OA also exacts significant costs from labour markets and from individuals through personal expenditures and reduced quality of life [6]. The prevalence of OA and its associated burden have increased in Canada year-over-year and are projected to continue increasing into the future due to population ageing and increasing obesity rates [7, 8].

There is no known cure or disease modifying therapy for knee and hip OA. First line management (pharmacological and non-surgical approaches) is therefore primarily focused on symptom mitigation and improving function in OA. When non-surgical management approaches fail, total knee or hip arthroplasty may be the only recourse for those with end-stage knee or hip OA. In addition to symptomatic control, the goal of current management approaches is also to possibly delay or prevent the need for arthroplasty through non-surgical treatment options [6, 9]. Patient education and exercise therapy are recommended non-surgical interventions for all patients with knee or hip OA across all major international treatment guidelines [10–14]. A recent systematic review found that treatment programs including structured education and exercise are cost-effective compared to usual physician-delivered care in numerous health settings [15], but underutilization of non-surgical interventions in general has been observed across various global healthcare settings [16–18]. Two recent Canadian studies have shown substantial underuse of education and exercise interventions in both people scheduled for total knee arthroplasty and in those not eligible for surgery [19, 20]. Thus, improved access to non-surgical care programs for people with knee and hip OA in Canada is needed.

To address the lack of available guideline-recommended non-surgical care for knee and hip OA, the Good Life with osteoArthritis in Denmark (GLA:D®) program was developed by researchers at the University of Southern Denmark [21]. GLA:D® consists of a two-day training course for health care providers, 8 weeks of a structured and supervised group education and exercise therapy program for patients, and a national clinical registry for program data collection [22]. By 2021 year-end, GLA:D® had expanded to 10 countries, with pilot projects in two additional countries, trained over 6,000 clinicians and had provided care for over 85,000 patients [23].

GLA:D® Canada was created and piloted in 2015, making it the first country outside Denmark to implement the program. The feasibility of implementing GLA:D® in Canada was demonstrated in a pilot study [24], and led to the national launch of GLA:D® Canada in 2017 by Bone and Joint Canada [25]. Since that time, programs like GLA:D® for knee and hip OA have been endorsed by multiple Canadian organizations as an economical means of improving care for people with knee or hip OA [26, 27]. Having now delivered care to Canadians for over 5 years, there is an opportunity to utilize the rich GLA:D® Canada clinical registry for enhancing OA research, following the lead of other national GLA:D® programs such as those in Denmark and Australia [28–33]. While brief introductory overviews of GLA:D® Canada participants and outcomes have been reported [25, 34], a comprehensive description of the GLA:D® Canada registry and participant characteristics has not been made available. Their availability is key to facilitating future research and research collaborations in this important area of care for a large clinical OA population. These data also offer the opportunity to learn about and improve health system delivery of OA care in Canada by making explicit comparisons with corresponding GLA:D® program data in multiple other health systems around the world.

The purpose of this report is to present the GLA:D® Canada registry and participant cohort to the Canadian and international OA research community by describing data collection procedures, health and outcome measures collected, and summarizing the sociodemographic and clinical profile of participants, with the overall intent to encourage collaborative research using these available data.

## Methods

### Design

This study report was a cross-sectional analysis of registry data. This report conforms to the STROBE statement for reporting observational studies [35]. Ethics approval for the GLA:D® Canada registry and this study were granted by the University Health Network Research Ethics Board (#16–5676). This study analysis did not involve any data that could identify individual participants.

### GLA:D® Canada program

**Fig 1:**
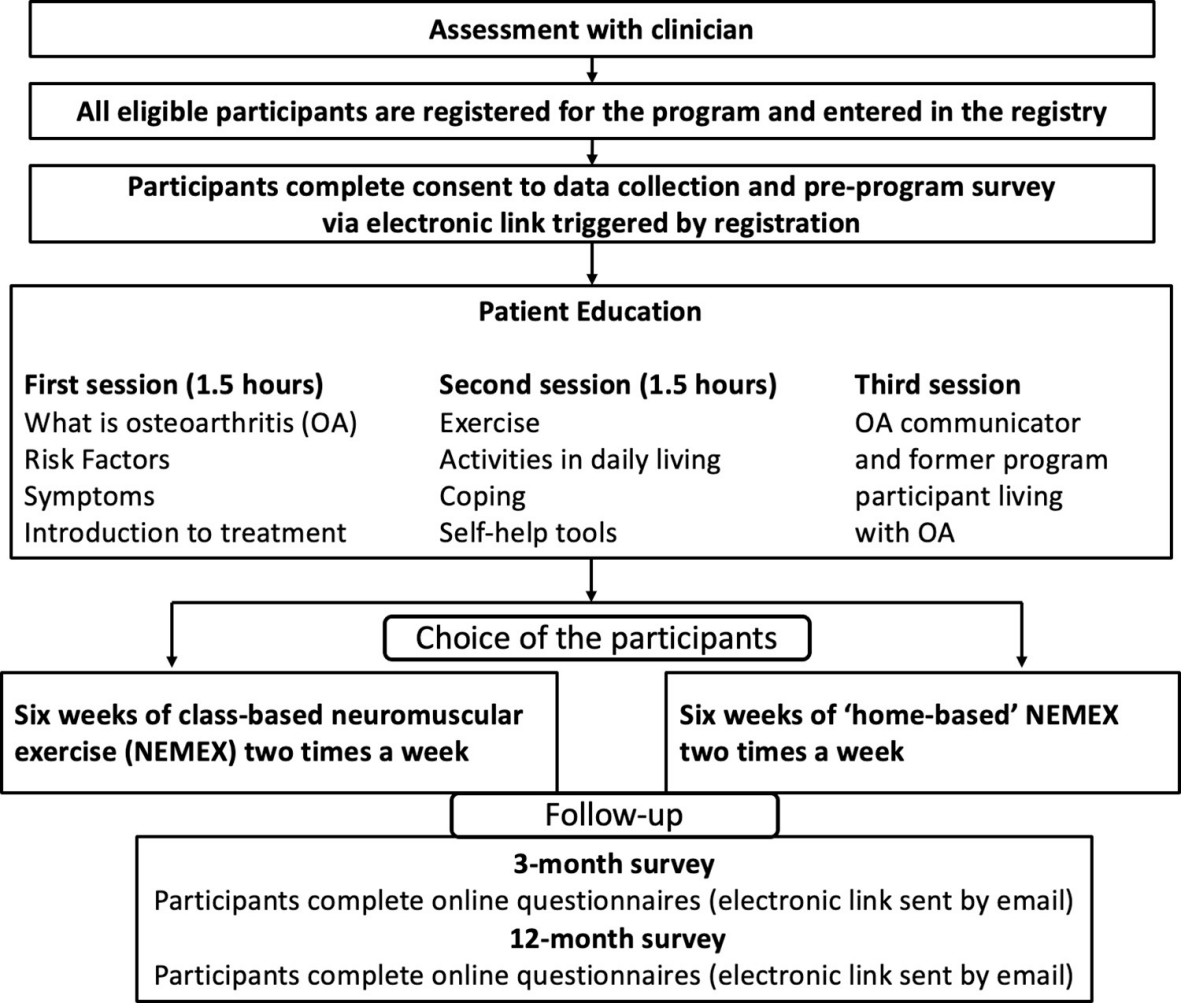
Overview of the GLA:D® Canada program.

Like all international GLA:D® programs, the GLA:D® Canada program for knee and hip OA consists of three standardized components: a training course for health care providers, a group-based patient education and exercise therapy program, and a patient data registry [22]. An overview of the GLA:D® Canada program and registry data collection are provided in Fig 1. All clinician and patient education materials were translated and adapted for the Canadian context as part of the 2015 feasibility study [24]. As of the end of 2022, 77 health care provider training courses had been held, licensing 2255 clinicians, primarily physiotherapists, to deliver GLA:D® across Canada. GLA:D® is currently offered in all provinces (except Quebec, where the program is not yet implemented) and two territories.

The group-based education and exercise therapy program consists of two patient education sessions (plus an optional third session) and 12 supervised exercise sessions, delivered twice a week [25]. Overall, the program is delivered over a six- to eight-week period. In the education sessions, GLA:D® clinicians lead discussions covering topics such as general knowledge about OA, symptoms, treatment and self-management options, and improving general health. An optional third patient education session lead by a former GLA:D® Canada participant and person with lived experience with OA may also be included. The educational material is continually updated to incorporate new evidence. The updates are led by the GLA:D® International Network to ensure material is standardized across international GLA:D® programs.

The clinician-supervised exercise sessions consist of a neuromuscular exercise program (NEMEX) aimed at promoting stabilization of the affected joints to help reduce pain and improve function [22, 36]. Detailed information on the content of the NEMEX program, including specific exercises, intensity levels and progressions, has been previously published [22, 30, 37]. Although the exercise sessions are standardized and delivered in a group-setting, the program is individualized for each participant by the supervising GLA:D® clinician based on ability to perform the different exercises and rate of progression over the program. There is also the option for participants to complete the exercise program at home if unable to participate in the group-based program at the clinic. Virtual program attendance is also a possibility at some clinics and was the only option at many clinics through the COVID-19 pandemic.

### Participants

Participants are eligible for GLA:D® if they have knee or hip joint problems as a result of OA that are sufficient in intensity to seek care in the health care system. Potential participants can access GLA:D® via referral from other health care providers or by self-referral to private clinics. There is no standardized method of how potential participants are informed about the program due to the varying provincial jurisdictions. Participants may be informed about the GLA:D® program by the general practitioner, physiotherapist, other patients, and/or via the GLA:D® Canada website (https://gladcanada.ca/).

An OA diagnosis is given by the enrolling GLA:D® clinician, using clinical signs and symptoms with no imaging requirement. While there are no specific clinical diagnostic criteria for knee or hip OA used by GLA:D® clinicians, patients are not eligible for the program if they have 1) joint problems related to other conditions (e.g. recent trauma, tumour, inflammatory disease, or sequelae after hip fracture); and 2) other symptoms that are more pronounced than OA symptoms (e.g. chronic, generalised pain, or fibromyalgia). These eligibility criteria are used by all international GLA:D® programs for OA [22, 30]. The GLA:D® Canada program is administered in English and French where available (since 2022).

Once participants are deemed eligible for the program, they are offered the ability to provide baseline and outcome data. Patients who consent to provide data are enrolled in the registry by the clinician. Patients who consent to provide data agree to make their data available for program evaluation and research purposes through the GLA:D® Canada registry. However, consent to provide data to the GLA:D® Canada registry is not required for participation in the GLA:D® program.

### Data collection procedures

GLA:D® clinicians register participants in the GLA:D® Canada registry and input information on the clinic where the program will be administered, participant index joint (knee or hip) for which they are seeking care, participant sex, health card number (optional), and conduct the objective functional tests (described below). All other data in the GLA:D® Canada registry are collected via electronic surveys sent directly to participants via the Data Driven Outcome System (DADOS). Electronic pre-program surveys are sent to patients at the time of registration by the GLA:D® clinician (prior to beginning the program) and then 3- (coinciding with the approximate end of the program) and 12-months after the pre-program survey date. Changes to the original 2016 electronic survey were made in 2019 and 2022 to include additional questions and measures.

### Objective physical function tests

Two tests, the 30-second chair stand test (repetitions) and 40-meter walk test (collected in seconds and converted to meters/second), are conducted by the GLA:D® clinician prior to the participant starting the program and written results are provided to the participant to be inputted into their electronic pre-program survey. These objective measures of physical function are recommended for use by the Osteoarthritis Research Society International as performance-based tests for people with knee and hip OA [38].

### Pre-program survey

#### Sociodemographic factors

Prior to starting the GLA:D® program, participants provide data on a number of sociodemographic factors, including: 1) age; 2) height and weight (used to calculate body mass index [BMI; m/kg^2^]); 3) current marital status (married/living with partner/single/divorced or separated/widowed); 4) highest level of education (elementary school/high school/trade or community college/university); and 5) current employment status (working full-time/working part-time/disability leave/unemployed/retired/other).

#### Clinical characteristics

Participants also provide data related to their medical status and clinical characteristics. Eleven medical comorbidities (yes/no), including cardiovascular disease, high blood pressure, high cholesterol, lung disease (asthma, chronic obstructive pulmonary disease or another chronic lung disease), diabetes (if yes, specific Type 1 or 2), kidney disease, liver disease, anemia or other blood disease, stomach or intestinal diseases (ulcers, gastritis, acid reflux, irritable bowel syndrome, Crohn’s disease), depression, cancer (within last five years, except minor cases of skin cancer), and rheumatological diseases (rheumatoid arthritis, fibromyalgia, or lupus) are collected and free-text fields are also available for participants to identify any other comorbidities not captured in the list. Clinical characteristics related to the index joint are collected, including: 1) duration of joint symptoms (years); 2) additional symptomatic knees and hips (yes/no for each knee and hip joint); 3) comorbid back pain (yes/no); 4) previous injury to the index joint (yes/no); 5) previous index joint surgery (yes/no); 6) desire for index joint surgery (yes/no); 7) current physical activity level (days per week); and 8) pain medication use (yes/no).

#### Health status measures

Knee or hip pain intensity in the last week is assessed using the Numeric Rating Scale (NRS), scored from 0 (no pain) to 10 (worst pain imaginable) [39]. Knee- or hip-related pain, function, and quality of life are assessed using the Knee injury and Osteoarthritis Outcome Score 12-item short form (KOOS-12) [40] or Hip disability and Osteoarthritis Outcome Score 12-item short form (HOOS-12) [41] subscales, respectively. All KOOS-12 and HOOS-12 subscales are scored from 0 (worst) to 100 (best). Health-related quality of life is assessed using the 5-level version of the EuroQol-5D (EQ-5D-5L) descriptive system (mobility, self-care, usual activities, pain/discomfort, anxiety/depression) [42], which is transformed to a utility score based on Canadian population weights [43]. EQ-5D-5L Utility scores range from -0.148 (worst health) to 0.949 (best health). The EQ-5D-5L Visual Analogue Scale (VAS) [42], scored 0 (worst health imaginable) to 100 (best health imaginable), is also collected. Arthritis management self-efficacy is assessed using the Arthritis Self-Efficacy Scale 8-item version (ASES-8), scored from 1 (low self-efficacy) to 10 (high self-efficacy) [44, 45].

#### Survey changes in 2019 and 2022

Six new questions and two additional measures were added to the pre-program survey in 2019. At the time of participant registration, GLA:D® clinicians are also asked to provide the payment source (private/public) for the patient. In the electronic patient survey, participants are also asked: 1) previous OA diagnosis by a health professional (yes/no/unsure); 2) currently waitlisted for surgery (yes/no); 3) problems walking due to their knee/hip problem (yes/no); 4) work leave in the past year due to knee/hip problem (yes/no); and 5) pain frequency in knee/hip (never/monthly/weekly/daily/always). Participants are also asked to complete the University of California at Los Angeles (UCLA) Activity Scale measuring their level of physical activity (1 low to 10 high) [46] and the Oxford Knee Score (OKS) or Oxford Hip Score (OHS) measuring overall pain and disability (0 worst to 48 best) [47].

Two new questions related to previous imaging of the index joint were added to the pre-program survey in 2022. Participants are also asked: 1) previous radiograph of knee/hip (yes/no); and 2) if yes, radiograph showed OA (yes/no/unsure).

### 3- and 12-month surveys

During the final exercise session, GLA:D® clinicians repeat the 30-second chair stand and 40-meter walk tests and give the written results to the participants to enter in their 3-month survey. These objective physical function tests are not recollected at the 12-month time-point. Other pre-program measures are recollected in both the 3- and 12-month surveys, including: pain NRS, KOOS-12 or HOOS-12, EQ-5D-5L, and ASES-8. The UCLA Activity Scale, OKS, and OHS were also added to both follow-up surveys in 2019. Other clinical questions such as pain medication use and desire for joint surgery are also asked in the follow-up surveys.

Participants are asked a set of additional questions related to patient experience during the program (i.e. not included in pre-program survey). In the 3-month survey only, participants are asked their level of satisfaction with GLA:D® (1 not at all satisfied to 5 very satisfied) and willingness to pay thresholds for GLA:D® ($100 or less to $301 or more). In both the 3- and 12-month surveys, participants are asked their level of benefit from the program (1 not at all beneficial to 5 very beneficial) and how often they use what they have learned in GLA:D® (never/every month/every week/every day/several times per day/don’t know). Since 2022, a 7-point scale comparing their current knee/hip problem to before the program (1 much better to 7 much worse) is also included in the 3- and 12-month survey.

### Data analysis

Patients in the GLA:D® Canada registry from program inception (2017) until 2022 year-end were included in this cross-sectional analysis of pre-program data. The number of participants who did not complete the pre-program survey were calculated but not included in the analysis. Data was accessed on 03 January 2023 for this analysis. The number of participants enrolled by year and proportions by geographical region since program inception were calculated. Pre-program characteristics of knee and hip participants were described separately. Proportions were reported for dichotomous and categorical data. The mean and standard deviation (SD) were reported for normally distributed continuous data. The median and inter-quartile range were reported for non-normally distributed continuous data. All data analyses were conducted in R version 4.2.1 (R Foundation for Statistical Computing).

## Results

**Fig 2:**
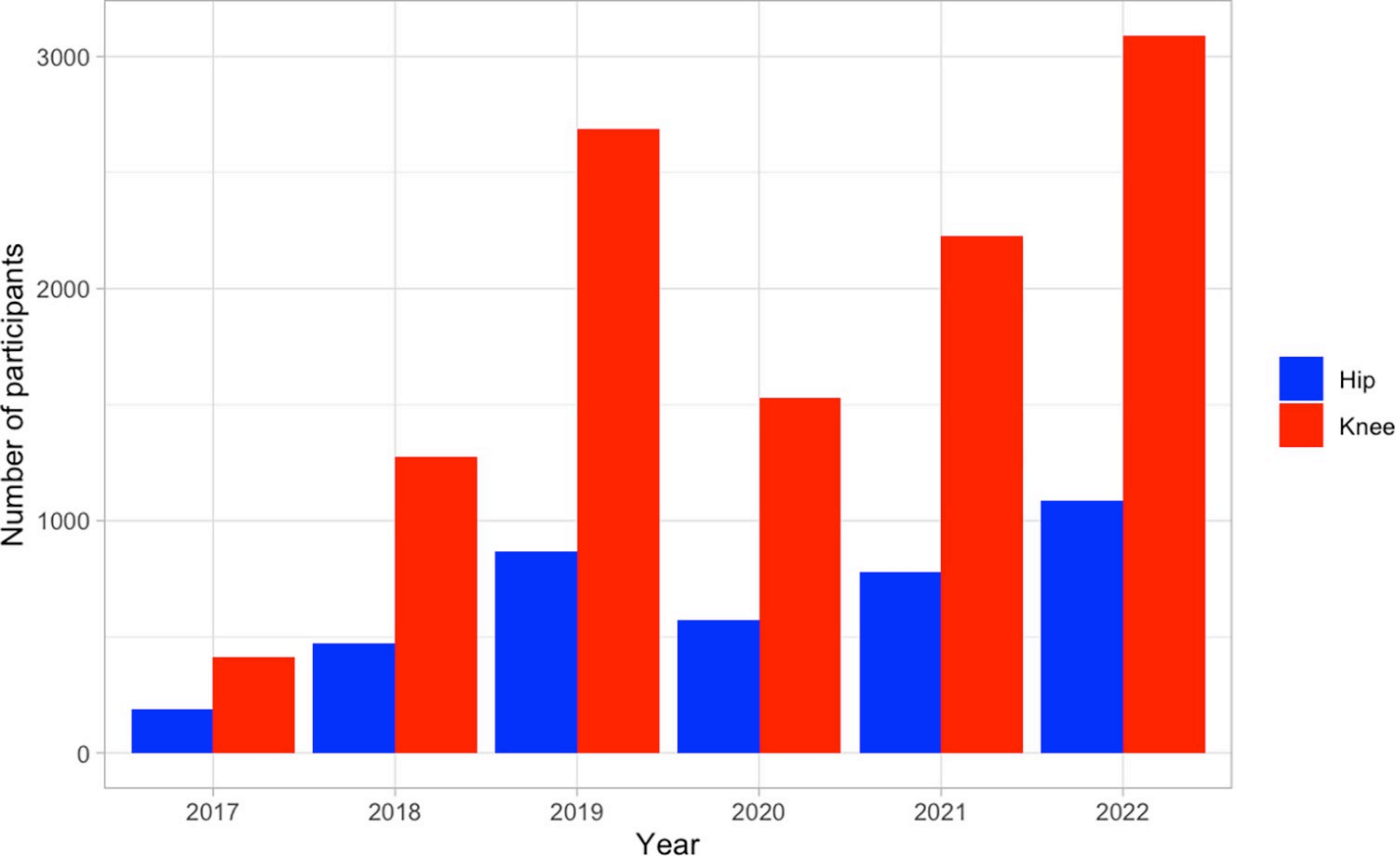
GLA:D® Canada enrolment per year from program inception in 2017 to 2022 year-end.

15,193 (11,228 knee; 3,965 hip) participants registered in the GLA:D® program from inception in 2017 to 2022 year-end (Fig 2). Participant registrations dropped in the 2020 and 2021 calendar years, reflecting the impact of mandated GLA:D® clinic closures due to the COVID-19 pandemic across Canada. In 2022, participant enrolment numbers surpassed pre-pandemic levels. Of those enrolled to date, 7,527 (67.0%) and 2,798 (70.6%) with knee and hip OA provided pre-program data. Table 1 presents the geographical reach of GLA:D® across Canada. Most participants (knee: 92.2%; hip: 91.5%) providing pre-program data were from the provinces of Ontario (knee: 61.2%; hip: 56.4%), Alberta (knee: 23.6%; hip: 27.3%), and British Columbia (knee: 7.4%; hip: 7.8%).

**Table 1:** Patient enrolment by region (province/territories) in Canada.

Region	Knee (n = 7,527)	Hip (n = 2,798)
Ontario	4,605 (61.2%)	1,579 (56.4%)
Alberta	1,779 (23.6%)	763 (27.3%)
British Columbia	555 (7.4%)	217 (7.8%)
New Brunswick	209 (2.8%)	87 (3.1%)
Nova Scotia	119 (1.6%	56 (2.0%)
Newfoundland	97 (1.3%)	22 (0.8%)
Manitoba	57 (0.8%)	26 (0.9%)
Saskatchewan	48 (0.6%)	23 (0.8%)
Prince Edward Island	42 (0.6%)	22 (0.8%)
Territories	16 (0.2%)	3 (0.1%)

Data presented as n (%).

### Participant characteristics

The profile of patients entering the program since inception are presented in Table 2. Thus far, GLA:D® participants are predominately female and, on average, are 66 years of age and obese (knee participants) or overweight (hip participants). Most participants are married, university-educated, and the two largest employment status groups are retired and full-time workers. Typically, participants have had knee or hip problems for years prior to GLA:D® (median of 4 years among knee participants, 3 years among hip participants) and have multiple symptomatic knee and hip joints. Most participants also have at least one medical comorbidity. About 1 in 3 participants reported a desire to have joint surgery before starting the program, while 22% of knee participants and 8% of hip participants have had a previous joint surgery. On average, participants are physically active four days per week, but a significant proportion do fear physical activity will damage their joints. Approximately 2 out of 3 participants are using pain medication at time of enrolment.

**Table 2:** Pre-program characteristics in knee and hip participants.

	Knee (n = 7,527)	Hip (n = 2,798)
Age (year)	65.7 (8.6)	65.9 (9.1)
Female	76.2%	74.4%
BMI (kg/m^2^)	30.7 (6.8)	28.6 (6.3)
Marital status Married Living with partner Single Divorced/separated Widowed	64.9% 6.2% 9.1% 10.9% 8.9%	65.3% 6.5% 8.7% 10.3% 9.2%
Education level Elementary school High school Trade or community college University	1.0% 15.2% 28.9% 54.9%	0.4% 14.2% 28.2% 57.3%
Employment status Working full-time Working part-time Disability leave Unemployed Retired Other	19.8% 8.7% 3.4% 1.7% 60.9% 5.5%	20.8% 9.1% 3.1% 1.2% 61.1% 4.7%
Number of comorbidities 0 1 2 3+	32.1% 28.8% 20.2% 19.0%	37.4% 29.3% 19.1% 14.3%
Symptom duration (years)*	4.0 (1.5 to 10.0)	3.0 (1.0 to 5.0)
Bilateral joint symptoms	63.2%	35.1%
Comorbid hip/knee symptoms	31.8%	51.7%
Back pain	19.5%	24.2%
Previous joint injury	43.2%	13.1%
Previous joint surgery	21.6%	8.0%
Desire for surgery	30.6%	35.3%
Physical activity level (days/week)	4.2 (2.2)	4.2 (2.2)
Fear physical activity will damage joints	31.9%	24.7%
Pain medication use	65.5%	68.0%
Pain NRS	5.1 (2.2)	5.2 (2.2)
K/HOOS-12 pain subscale	51.1 (15.9)	50.3 (16.6)
K/HOOS-12 function subscale	56.3 (19.5)	57.0 (19.3)
K/HOOS-12 quality of life subscale	38.8 (17.6)	41.8 (19.1)
EQ-5D-5L utility score*	0.7 (0.7 to 0.8)	0.7 (0.6 to 0.8)
EQ-5D-5L VAS*	72.0 (60.0 to 80.0)	70.0 (60.0 to 80.0)
ASES-8	6.1 (1.8)	5.9 (1.8)
30-second chair stand test (repetitions)	11.9 (5.2)	12.3 (5.2)
40-meter walk test (m/s)	1.3 (0.4)	1.3 (0.4)

Data presented as means (SD) or %, except where * indicates median (IQR) reported because non-normal distribution. NRS = Numeric Rating Scale (0 no pain to 10 worst pain imaginable); KOOS-12 = Knee injury and Osteoarthritis Outcome Score 12-item short form (all subscales 0 worst to 100 best); HOOS-12 = Hip disability and Osteoarthritis Outcome Score 12-item short form (all subscales 0 worst to 100 best); EQ-5D-5L = EuroQol-5D 5-level version (-0.148 worst to 0.949 best); EQ-5D-5L VAS = EQ-5D-5L Visual Analogue Scale (0 worst health imaginable to 100 best health imaginable); ASES-8 = Arthritis Self-Efficacy Scale 8-item version (1 low self-efficacy to 10 high self-efficacy).

The average pain intensity (pain NRS) rating is 5 out of 10 for both knee and hip participants. Mean scores for knee- and hip-related pain (K/HOOS-12 pain subscale), function (K/HOOS-12 function subscale), and quality of life (K/HOOS-12 quality of life subscale), health-related quality of life (EQ-5D-5L Utility and VAS), and self-efficacy in managing their OA were likewise similar for participants with knee and hip OA. Knee and hip participants could complete an average of 12 rises in the 30-second chair stand test and had an average walking speed of 1.3 m/s on the 40-meter walk test.

Pre-program characteristics for the new data captured effective 2019 and 2022 are presented in Table 3. Since 2019, over half of participants accessed GLA:D® via public funding. Over 90% of participants had a previous diagnosis of OA from a health care professional, while approximately one in 10 participants were on a surgical waitlist at time of program enrolment. Most GLA:D® participants reported experiencing daily or constant pain, difficulty walking, and can sometimes participate in moderate physical activities based on mean UCLA Activity scores. The OKS and OHS mean scores suggest, on average, that participants experience moderate severity knee and hip OA. Almost all participants since the beginning of 2022 reported having had a previous radiograph of their joint, with most reporting the radiograph had findings associated with OA.

**Table 3:** Pre-program characteristics of knee and hip participants included in pre-treatment survey updates since 2019.

	Knee	Hip
**Included since 2019 survey update**	**n = 3,412**	**n = 1,265**
Payment source Private Public	40.8% 59.2%	44.7% 55.3%
OA diagnosis Yes No Unsure	90.4% 3.7% 5.9%	91.4% 3.6% 5.0%
Waitlisted for surgery	8.6%	11.7%
Problems walking	77.7%	80.8%
Work leave in past year	10.2%	8.2%
Pain frequency Never Monthly Weekly Daily Always	1.5% 2.9% 11.4% 62.3% 21.8%	2.3% 2.1% 10.0% 62.3% 23.2%
UCLA Activity Scale	5.1 (1.7)	5.0 (1.7)
Oxford Knee/Hip Score	30.3 (7.8)	29.7 (8.4)
**Included since 2022 survey update**	**n = 1,567**	**n = 577**
Previous radiograph	92.0%	90.2%
Radiograph showed signs of OA Yes No Unsure	86.8% 2.1% 11.1%	87.6% 1.6% 10.9%

Data presented as means (SD) or %. UCLA Activity Score = University of California at Los Angeles (UCLA) Activity Scale (1 low to 10 high); Oxford Knee/Hip Score (0 worst to 48 best).

## Discussion

The GLA:D® Canada registry comprises one of the largest datasets with a comprehensive set of characteristics and measures from a primary care cohort of patients with knee or hip OA seeking care in a nation-wide patient education and exercise therapy program. Here we have provided detailed information on the data collection procedures and participant profiles from this large cohort, with a goal of encouraging research collaborations that will make use of these data to advance OA research and improve OA care.

Our findings show that GLA:D® Canada participants with knee or hip OA are remarkably similar to participants in the GLA:D® Denmark and Australia programs [22, 30, 34]. Across all three programs, the majority of participants are female and present with a primary complaint of knee problems. Likewise, the average age is approximately 66 years, average BMI is in the overweight or obese range, and a similar proportion of participants report pain medication use and having had a previous joint surgery. Furthermore, outcome measure scores are similar across the three international GLA:D® programs: pain NRS scores around 5 out of 10, 30-second chair stand test scores around 12 repetitions, and 40-meter walk test speeds around 1.5 m/s [34]. This presents remarkable opportunities for bridging international datasets. We are unable to compare scores based on the HOOS/KOOS (knee/hip-related pain, function and quality of life) as the Danish and Australian programs use the full HOOS/KOOS versions (compared to 12-item KOOS-12 and HOOS-12 versions used in Canada). However, a 2019 analysis of GLA:D® data from these three countries (the full HOOS/KOOS versions were collected in Canada until the 2019 survey update) found Canadian participants had slightly worse pre-program quality of life scores [34]. It is unclear if the findings from this smaller Canadian sample (1,182 combined knee and hip participants) are similar to the larger cohort now available. The considerable overlap in findings between individuals with OA participating in the GLA:D® Canada program with those from more established programs like GLA:D® Denmark and GLA:D® Australia bodes well for international research collaborations investigating implementation and impact outcomes for education and exercise programs for individuals with hip and knee OA.

### Future research

The GLA:D® Canada registry was designed with the intent to address OA research questions that consider patients or the impact of a program in a real-world clinical setting. It is the hope that making these already collected data available to the larger research community will foster opportunities for primary and collaborative OA research, for trainees to established investigators, with the ultimate goal of improved care and quality of life for the millions of individuals living with hip and knee OA. There are many examples of the use of international GLA:D® datasets by researchers undertaking OA-related investigations [30, 32–34, 48–53], including qualitative investigations in people with OA and/or health care providers [31, 54, 55]. Furthermore, participant data in the GLA:D® Canada registry can be linked via Personal Health Number to provincial public administrative health systems data, such as from the Alberta Health Care Insurance Plan or Ontario Health Insurance Plan, offering the opportunity to examine health care utilization among GLA:D® participants. For example, it would be prudent to investigate if participation in GLA:D® is related to reduced need for total joint replacement surgery. Finally, the GLA:D® Canada program exists within a large network of international GLA:D® programs for knee and hip OA and low back pain, all of which share a certain level of common data collection. Insights from comparisons across global jurisdictions could help to improve health service delivery for OA across Canadian provincial and territorial health systems. Researchers with an interest in the global perspective are encouraged to join and build upon previous international collaborations within the GLA:D® International Network [34].

### Limitations

The lack of specific eligibility criteria in GLA:D®, namely the lack of requirement for radiologically-defined OA, can raise questions about participants truly having knee or hip OA. Despite the weak correlation between patient symptoms and imaging findings reported in the literature and international consensus that radiographic evidence is no longer needed to diagnose knee or hip OA [6, 9, 56–58], the notion of OA as an imaging-based disease still persists. GLA:D® Canada, like all GLA:D® programs, relies on the enrolling GLA:D® clinicians to determine if their patients are eligible for the program via clinical examination. All GLA:D® programs for knee or hip OA involve a health care provider training course where the most recent diagnostic literature is presented and clinical diagnostic criteria are discussed. While comparable data from GLA:D® Canada are not available, analyses from GLA:D® Denmark show the vast majority of knee and hip patients satisfy major international diagnostic criteria, including 89% and 94% fulfilling the National Institute for Health and Care Excellence criteria for knee and hip OA, respectively [32, 48]. These findings suggest that the health care provider training program sufficiently prepares GLA:D® clinicians to identify knee and hip OA. We have provided further supporting evidence in this report, where since the time of specific data inclusion in the pre-program survey, the vast majority (>90%) of GLA:D® Canada participants report having been given an OA diagnosis in their index joint by a previous health care provider and have had images suggesting OA in that joint. Therefore, researchers can be confident the GLA:D® Canada registry contains data from people seeking care for knee or hip OA.

The strengths of a real-world and national patient cohort registry come with some trade-offs. The program is administered in and registry data are collected from numerous clinical settings, unlike a highly controlled research setting. This may give rise to concerns about treatment fidelity, selection bias due to voluntary enrolment in the program and registry, and the equal application of patient eligibility and enrolment criteria across all clinical sites. Likewise, completion of the data collection surveys is not mandatory, although highly encouraged, for participation in the GLA:D® Canada program, which could lead to suboptimal data completion. For example, approximately 1 in 3 GLA:D® participants in this cohort did not provide pre-program data once registered and enrolled. Unfortunately, no data is available to enable comparisons of those who do and do not complete the pre-program survey. For the same reasons, missing data is also a risk at follow-up time-points. A thorough program evaluation would help to clarify potential concerns and highlight areas for improvement in the implementation process. Despite these limitations, the GLA:D® Canada registry offers the unique opportunity to conduct research on a large sample of patients managed in real-world clinical settings.

## Conclusion

The GLA:D® Canada registry contains detailed data on participant profiles, across a wide range of measures, for two large cohorts of patients seeking care for knee or hip OA, respectively. Patient enrolment continues in these programs and treatment outcome data are also available. Through their broad availability, researchers are invited to make use of these rich datasets and participate in collaborative endeavours to tackle questions of Canadian and global importance for a large and growing clinical population of individuals with hip and knee OA.

## Supporting information

S1 Checklist STROBE statement—checklist of items that should be included in reports of observational studies. (DOCX)
